# Gentiopicroside promotes the osteogenesis of bone mesenchymal stem cells by modulation of β‐catenin‐BMP2 signalling pathway

**DOI:** 10.1111/jcmm.16410

**Published:** 2021-11-15

**Authors:** Huaji Jiang, Jialiang Zhong, Wenjun Li, Jianghui Dong, Cory J Xian, Yung‐Kang Shen, Lufeng Yao, Qiang Wu, Liping Wang

**Affiliations:** ^1^ Department of Orthopaedic Yuebei People's Hospital Affiliated to Medical College of Shantou University Shaoguan China; ^2^ Department of Immunology School of Basic Medical Science Southern Medical University Guangzhou China; ^3^ Department of Clinical Laboratory The Seventh Affiliated Hospital Sun Yat‐sen University Shenzhen China; ^4^ UniSA Clinical& Health Sciences University of South Australia Adelaide SA Australia; ^5^ School of Dental Technology College of Oral Medicine Taipei Medical University Taipei Taiwan; ^6^ Department of Foot and Ankle Surgery Ningbo No. 6 Hospital Ningbo China

**Keywords:** BMP pathway, Gentiopicroside, osteogenic factors, osteogenic parameters, osteoporosis, Wnt/β‐catenin pathway

## Abstract

Osteoporosis is characterized by increased bone fragility, and the drugs used at present to treat osteoporosis can cause adverse reactions. Gentiopicroside (GEN), a class of natural compounds with numerous biological activities such as anti‐resorptive properties and protective effects against bone loss. Therefore, the aim of this work was to explore the effect of GEN on bone mesenchymal stem cells (BMSCs) osteogenesis for a potential osteoporosis therapy. In vitro, BMSCs were exposed to GEN at different doses for 2 weeks, whereas in vivo, ovariectomized osteoporosis was established in mice and the therapeutic effect of GEN was evaluated for 3 months. Our results in vitro showed that GEN promoted the activity of alkaline phosphatase, increased the calcified nodules in BMSCs and up‐regulated the osteogenic factors (Runx2, OSX, OCN, OPN and BMP2). In vivo, GEN promoted the expression of Runx2, OCN and BMP2, increased the level of osteogenic parameters, and accelerated the osteogenesis of BMSCs by activating the BMP pathway and Wnt/β‐catenin pathway, effect that was inhibited using the BMP inhibitor Noggin and Wnt/β‐catenin inhibitor DKK1. Silencing the β‐catenin gene and BMP2 gene blocked the osteogenic differentiation induced by GEN in BMSCs. This block was also observed when only β‐catenin was silenced, although the knockout of BMP2 did not affect β‐catenin expression induced by GEN. Therefore, GEN promotes BMSC osteogenesis by regulating β‐catenin‐BMP signalling, providing a novel strategy in the treatment of osteoporosis.

## 1 INTRODUCTION

Osteoporosis is a systemic disease characterized by bone loss, destruction of the bone microstructure and increased bone fragility, often leading to brittle fractures.^1^ A large number of patients with osteoporosis not only suffer from severe pain, but they are also subjected to a heavy financial burden.^2^ In particular, the bone resorption rate of postmenopausal women is significantly higher than that of osteogenesis, leading to a serious bone loss.^3^ With the development of ageing population, the number of postmenopausal patients with osteoporosis is increasing year by year, causing a significant impact on the medical community and the whole society.^4^ At present, the drugs used to treat postmenopausal osteoporosis play a role mainly by inhibiting bone absorption and promoting bone formation.^5^, ^6^ However, long‐term use of anti‐osteoporosis drugs can cause a series of adverse reactions, including myasthenia gravis, influenza‐like diseases and gastrointestinal tumours.^7^ Thus, it is of utmost importance to find a new alternative therapy to cure osteoporosis. The activity of osteoclasts and osteoblasts needs to be precisely coordinated to maintain skeletal integrity.^8^ Osteoclasts are multinucleated giant cells derived from monocytes/macrophages, whose main function is to promote bone resorption.^9^ Osteoblasts are mainly differentiated from bone mesenchymal stem cells (BMSCs) and deposited in the calcified bone matrix, which has a significant impact on the formation of a new bone.^10^ The primary cause of osteoporosis is due to the decrease in osteoblasts that leads to a reduced bone formation, and the increase in osteoclasts resulting in an increased osteolysis. Recently, several reports pointed out that stimulating osteoblast differentiation may be an effective way to prevent and treat osteoporosis.^11^, ^12^, ^13^

BMSCs are stem cells with a multi‐directional differentiation, as they can differentiate into several cell types including chondrocytes, osteoblasts, adipocytes and endothelial cells under specific conditions.^14^ BMSCs first differentiate into precursor osteoblasts, then into osteoblasts, and finally they gradually form the mature osteoblasts.^15^ However, the osteogenic capability of BMSCs gradually decreases with the increase of old age, whereas the adipogenic capacity of BMSCs increased, leading to the down‐regulation of bone formation and finally osteoporosis.^16^ During the osteogenic differentiation, BMSCs release a series of osteogenic factors, including osteocalcin (OCN), osterix (OSX), osteopontin (OPN) and runt‐related transcription factor 2 (Runx2), which accelerate the maturation of osteoblasts.^16^, ^17^ Therefore, BMSCs may be a suitable cell source for studying osteogenesis.

The Wnt pathway and BMP pathway occupy a critical position in the modulation of osteoblast differentiation.^18^, ^19^ When the frizzled transmembrane receptor binds to LRP5 and/or LRP6, it can induce the secretion of Wnts and activates the canonical Wnt pathway.^20^ Subsequently, β‐catenin is released and transcribed into the nucleus to regulate the generation of osteogenic markers.^21^ The up‐regulation of BMP pathway induces the phosphorylation of Smad proteins.^22^ Then, the activated Smad proteins actively regulate the transcription of osteogenic factors (Runx2 and OCN), thus promoting osteogenic differentiation.^23^, ^24^

GEN can be obtained from the natural plant *Gentiana ManshuricaKitag*, and it is a type of natural compounds with numerous biological activities such as anti‐resorptive properties. It is widely used in the traditional Chinese medicine. GEN has a variety of pharmacological activities, including antioxidative, anti‐inflammatory, antibacterial and anti‐osteoporotic effects.^25^, ^26^, ^27^ Recently, it was reported that GEN suppressed RANKL‐induced osteoclastogenesis by regulating NF‐κB and JNK signalling pathways and that it represented a potential drug in the treatment of osteoporosis.^28^Based on the above studies, our hypothesis is that GEN could induce osteogenic differentiation of BMSCs in vitro and bone formation in vivo.

Collectively, in order to understand the mechanism of action of GEN during bone formation, the effect of GEN on BMSCs function needs to be further characterized. Therefore, the purpose of this study was to find whether GEN could promote the osteogenic differentiation of BMSCs and explore the molecular mechanism induced by GEN in differentiating BMSCs. We used the ovariectomized (OVX) mouse model, the most popular animal model for postmenopausal osteoporosis, in which the acceleration of cancellous bone loss and the decrease of cortical bone are closely correlated to oestrogen deficiency.^17^, ^29^ Thus, this model is beneficial for us to explore the influence of GEN on bone density and osteogenic factor expression in vivo.

## 2 MATERIALS AND METHODS

This project was carried out with the permission of the Ethics Committee of the Ningbo No. 6 Hospital (registered number 2015‐018).

### 2.1 Cell culture and treatments

Five female C57BL/6 mice (4‐week‐old) were used and killed to obtain BMSCs cultured according to a previous work.^30^ In brief, the mouse femur was collected and the surrounding soft tissues were removed. Then, the bone marrow in the femur was washed three times with α‐MEM (Sigma‐Aldrich). The obtained bone marrow content was placed in a dish containing a complete medium(Sigma‐Aldrich). BMSCs were incubated at 37°C in a 5% carbon dioxide incubator. The third generation of BMSCs was used for our experiments. When the confluence reached 70%, the osteogenic medium was added to allow the osteogenic differentiation. The osteogenic medium consisted of complete medium supplemented with 0.1 mM dexamethasone (Sigma‐Aldrich), 5 mM β‐glycerophosphate (Sigma‐Aldrich) and 100 mM ascorbic acid (Sigma‐Aldrich). Then, BMSCs were cultured for 14 days under the osteogenic environment, changing the medium every 3 days.

The silencing of β‐catenin and BMP 2 gene was performed according to previous investigations.^31^, ^32^ The transfection sequences were the following: *β‐catenin*, former primer 5’‐AAGGTAGAGTGATGAAAGTTGTT‐3’ and reverse primer 5’‐CACCATGTCCTCTGTCTATTC‐3’. *BMP2*, former primer 5’‐AGGGTTTCAGGTCAGTTTCCG‐3’ and reverse primer 5’‐GATGATGAGGTTCTTGGCGG‐3’. The cDNA of β‐Catenin and BMP2 were transferred into ad lentivirus of Cre recombinase (Ad‐Cre; System Biosciences, Hercules, CA, USA). BMSCs at a density of 2.08 × 10^4^ cells/cm^2^ were seeded into 6‐well plates for 24 hours. Next, the cells cultured in the osteogenic medium and transferred with the Ad‐Cre (at a concentration of 5 × 10^8^ pfu/mL) for 48 hours. Ad‐GFP was used as a control. Next, the cells were treated with 40 μM GEN in the osteogenic medium for 7 days. Finally, the expression of Runx2, OSX, OCN, OPN, BMP2 and β‐catenin was evaluated by q‐PCR and Western blotting.

### 2.2 Cell cytotoxicity test

BMSCs (3.13 × 10^4^ cells/cm^2^) were seeded into 96‐well plates and cultured for 24 hours. Then, GEN at different concentrations (0, 10, 20, 40 and 80 μM) was added, and the cells were cultured for 7 days. At the end of the 7 days, the Cell Counting Kit‐8 (CCK‐8; Sigma‐Aldrich) was used to evaluate the cytotoxicity of GEM. The absorbance was measured at 450 nm according to the manufacturer recommendations. A previous study described this method in detail.^29^

### 2.3 Alkaline phosphatase activity measurement

BMSCs (2.08 × 10^4^ cells/cm^2^) were seeded into 6‐well plates and then treated with GEN at different concentrations (0, 10, 20 and 40 μM) for 14 days under osteogenic environment. Next, BMSCs were washed with medium for three times and lysed by ultrasound. The concentration of the lysate protein was measured by the Bradford protein test (Thermo Fisher Scientific, Waltham, MA). Alkaline phosphatase (ALP) activity was detected using p‐nitrophenyl phosphate in AMP buffer (Sigma‐Aldrich) at room temperature for 20 minutes. Then, sodium phosphate (0.3 M, pH 12.3, Sigma) was used to terminate the reaction. Finally, the results of ALP activity were standardized according to the protein concentration. Next, BMSCs were fixed with formalin for 10 minutes, ALP staining buffer (Sigma‐Aldrich) was added, and the cells were incubated for 30 minutes at room temperature.

### 2.4 Alizarin red staining

BMSCs (2.08 × 10^4^ cells/cm^2^) were seeded into 6‐well plates and treated with GEN at different concentrations (0, 10, 20 and 40 μM) for 14 days under osteogenic environment. Next, the medium was discarded, the cells were washed with PBS for three times, and a formalin solution was added to fix the cells. After 20 minutes, the formalin was discarded, and BMSCs were washed 2 times with medium and treated with alizarin red solution (Sigma‐Aldrich) for 30 minutes. Subsequently, the stained cells were observed under an optic microscope and images were taken in random fields. Finally, the software Image J (NIH, Bethesda, MA, USA) was used to perform the statistics of the mineralized nodules, and the nodules larger than 0.04 mm were included in the statistical calculation.^33^

### 2.5 Quantitative PCR (q‐PCR)

BMSCs (2.08 × 10^4^ cells/cm^2^) were seeded into 6‐well plates and treated with GEN at different concentrations (0, 10, 20 and 40 μM) in the osteogenic medium for 14 days. Then, the mRNA expression of *ALP*, *OPN*, *OCN*, *OSX*, *Runx2* and *BMP2* was detected by q‐PCR. Trizol reagent (Sigma‐Aldrich) was used to extract the RNA from BMSCs. The total RNA was translated into cDNA according to the reverse‐transcribed kit (Applied Biosystems, USA) using the following parameters: 95°C for 9 minutes, 36°C for 40 minutes for 2 cycles, then 86°C for 4 minutes, and final cooling to 4°C. The cDNA of the target gene was quantified by q‐PCR using the SYBR Green Premix kit (Roche, Switzerland). The q‐PCR parameters were the following: 95°C for 20 seconds, 90°C for 10 seconds for 40 cycles, and 60°C for 30 seconds. The primers used in this study (Life Technologies) were the following: *ALP*, forward primer AACCCAGACACAAGCATTCC, reverse primer GAGAGCGAAGGGTCAGTCAG *Runx2*, forward primer AATTAACGCCAGTCGGAGCA, reverse primer CACTTCTCGGTCTGACGACG *OSX*, forward primer CACTTCTCGGTCTGACGACG, reverse primer CACTTCTCGGTCTGACGACG *OCN*, forward primer CACTTCTCGGTCTGACGACG, reverse primer ATAGCTCGTCACAAGCAGGG *BMP2*, forward primer GCTTCCGTCCCTTTCATTTCT, reverse primer GCTTCCGTCCCTTTCATTTCT *OPN*, forward primer GCTTCCGTCCCTTTCATTTCT, reverse primer GCTTCCGTCCCTTTCATTTCT *GAPDH*, forward primer CATCACTGCCACCCAGAAGAC, reverse primer CCAGTGAGCTTCCCGTTCAG. GAPDH was used as the internal control. The relative gene expression was calculated using the 2^‐ΔΔCt^ method.

### 2.6 Western blot

BMSCs (2.08 × 10^4^ cells/cm^2^) were seeded into 6‐well plates and treated with GEN at different concentrations (0, 10, 20 and 40 μM) in the osteogenic medium for 14 days. To assess the influence of GEN on the signalling of BMP and Wnt/β‐catenin, BMSCs were treated with either 300 ng/mL Noggin (Sigma‐Aldrich)^34^ or 100 ng/ml DKK‐1 (Sigma‐Aldrich)^35^ in the treatment with GEN for 2 weeks. After the treatment, the total BMSC proteins were extracted by radioimmunoprecipitation assay lysis buffer (Sigma‐Aldrich) at 4°C for 30 minutes. After centrifugation, the supernatant was collected, and the proteins were separated by electrophoresis and transferred to a PVDF membrane according to a previous study (Xiao et al 2015). The primary antibodies used in this work were the following: anti‐BMP‐2 (1:2000; Sigma‐Aldrich), anti‐β‐catenin (1:1500; Cell Signalling Technology), anti‐phospho‐Smad1/5/8 (1:5000; Sigma‐Aldrich) anti‐β‐actin (1:3000; Cell Signalling Technology), anti‐Runx2 (1:800; Cell Signalling Technology) and anti‐OCN (1:800; Cell Signalling Technology). Next, the PVDF membrane was washed by TBST thrice, each time for 5 minutes, and the second antibody goat anti‐rabbit (1:3000, Abcam) was added. The specific protein bands were visualized using a proprietary chemiluminescence kit (PerkinElmer, Inc, Waltham, MA). The bands were quantified by densitometry using the Image‐Pro Plus 6.0 software (Media Cybernetics, Rockville, MD). The target proteins were first corrected over β‐actin expression and then as fold change from control group.

### 2.7 Experimental model and animal groups

Thirty‐six female C57BL/6 mice (8‐week‐old, 21 ± 2 g) were obtained from the animal experimental centre of the Southern Medical University (Guangzhou, Guangdong, China). The experimental mice were stochastically divided into three groups: Sham group (n = 12), ovariectomized (OVX) group (n = 12) and OVX + GEN group (n = 12). The OVX + GEN groups were treated by an oral gavage of 50 mg/kg/day GEN during these 3 months. The Sham group and OVX group received the same dose of saline by oral gavage. After 3 months, the experimental mice were killed by cervical dislocation and the femurs were collected for further studies.

### 2.8 Histological and immunohistochemical staining

The femur was immersed in 4% paraformaldehyde for 48 hours and decalcified using 15% ethylenediaminetetraacetic acid for 14 days. Then, they were dehydrated, paraffin embedded and cut into 4‐μm‐thick sections. To perform the haematoxylin‐eosin (HE) staining, the sections were dewaxed, hydrated and stained with HE dyes (Abcam). Finally, the HE‐stained sections were photographed and analysed. As regard the immunohistochemistry, the sections were dewaxed, hydrated treated with 3% hydrogen peroxide for 15 minutes and with protease K for 10 minutes. Next, the sections were treated with primary antibodies and incubated overnight at 4°C. The primary antibodies (Santa Cruz Biotechnology) used were the following: anti‐Runx2 (1:200), anti‐BMP‐2 (1:200) and anti‐OCN (1:200). The sections were washed with PBS thrice for a total of 15 minutes, and the second antibody was added and incubated for 50 minutes. Next, the sections were washed three times with PBS, and then, diaminobenzidine solution was added to obtain the chromogenic reaction. Finally, the sections were observed and analysed under an optical microscope.

### 2.9 Microcomputer tomography analysis

The collected femurs were preserved in 4% paraformaldehyde for 48 hours. The prepared femur was scanned and analysed by high‐resolution micro‐CT (Caskaisheng, China). The scanning parameters of the micro‐CT were set as follows: 80 kV, 15 μA and a scanning thickness of 20 μm. The area below the crud end of femoral shaft was chosen as the analysis area for statistical analysis.^36^ The bone parameters for statistical analysis included the following three indexes: trabecular bone mineral density (BMD), trabecular number and trabecular thickness.

### 2.10 Statistical analysis

Statistical analysis was performed using GraphPad Prism 6 (Manufacturer, La Jolla, CA, USA). All in vitro experiments were repeated three times, and each experiment was carried out in triplicate. In the in vivo experiments, each group contained at least 6 rats. Results were expressed as mean ± standard deviation (SD). One‐way ANOVA and Dunnett's test were used to compare multiple groups, whereas unpaired Student's t test was used for the comparison of two groups. *P* <.05 was considered statistically significant.

## 3 RESULTS

### 3.1 Effect of GEN on BMSC proliferation

**FIGURE 1:**
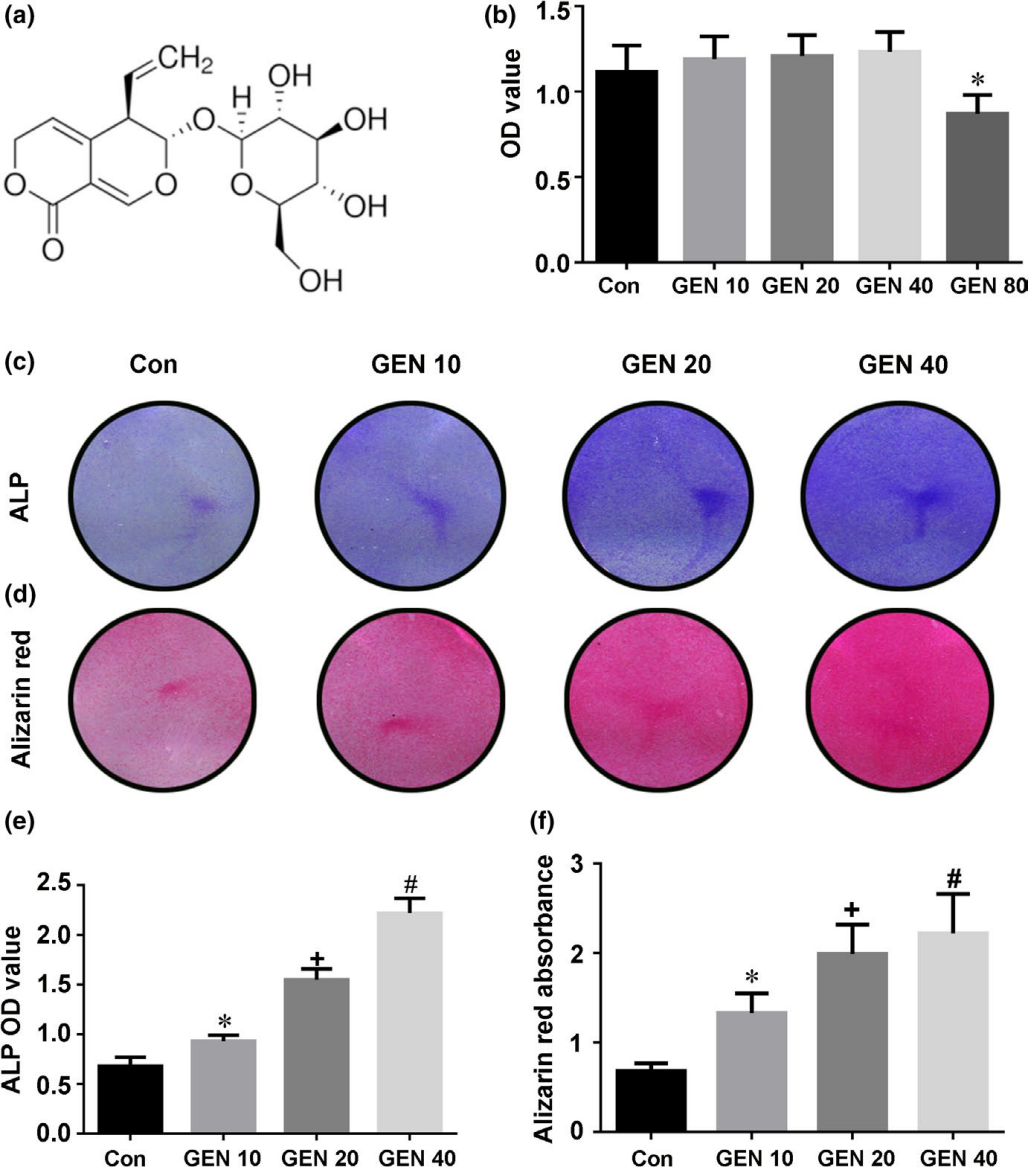
Effect of GEN on the proliferation and differentiation of BMSCs. A, Chemical structure of GEN. B, BMSCs were cultivated with increasing doses of GEN (0, 10, 20, 40 and 80 μM) for 1 week. The proliferation of BMSCs was evaluated by CCK‐8 test. BMSCs were cultivated with GEN (0, 10, 20 and 40 μM) in the condition of osteogenic induction for 2 weeks. The activity of ALP was tested by ALP staining (C, E), whereas mineralized nodules were detected by Alizarin Red staining (D, F). *P <.05 versus group without GEN, ^+^ *P* <.01 versus 10 μM GEN, ^#^ *P* <.001 versus 20 μM GEN

The chemical structure of GEN is shown in Figure 1A. The proliferation of BMSCs treated with GEN (10‐40 μM) was not significantly changed (Figure 1B). However, 80 μM GEN significantly inhibited the proliferation of BMSCs (less than 1‐fold, *P* <.05). Thus, GEN was not harmful to BMSCs at the concentrations of 10, 20 and 40 μM.

### 3.2 GEN strengthens the osteogenic differentiation in BMSCs

**FIGURE 2:**
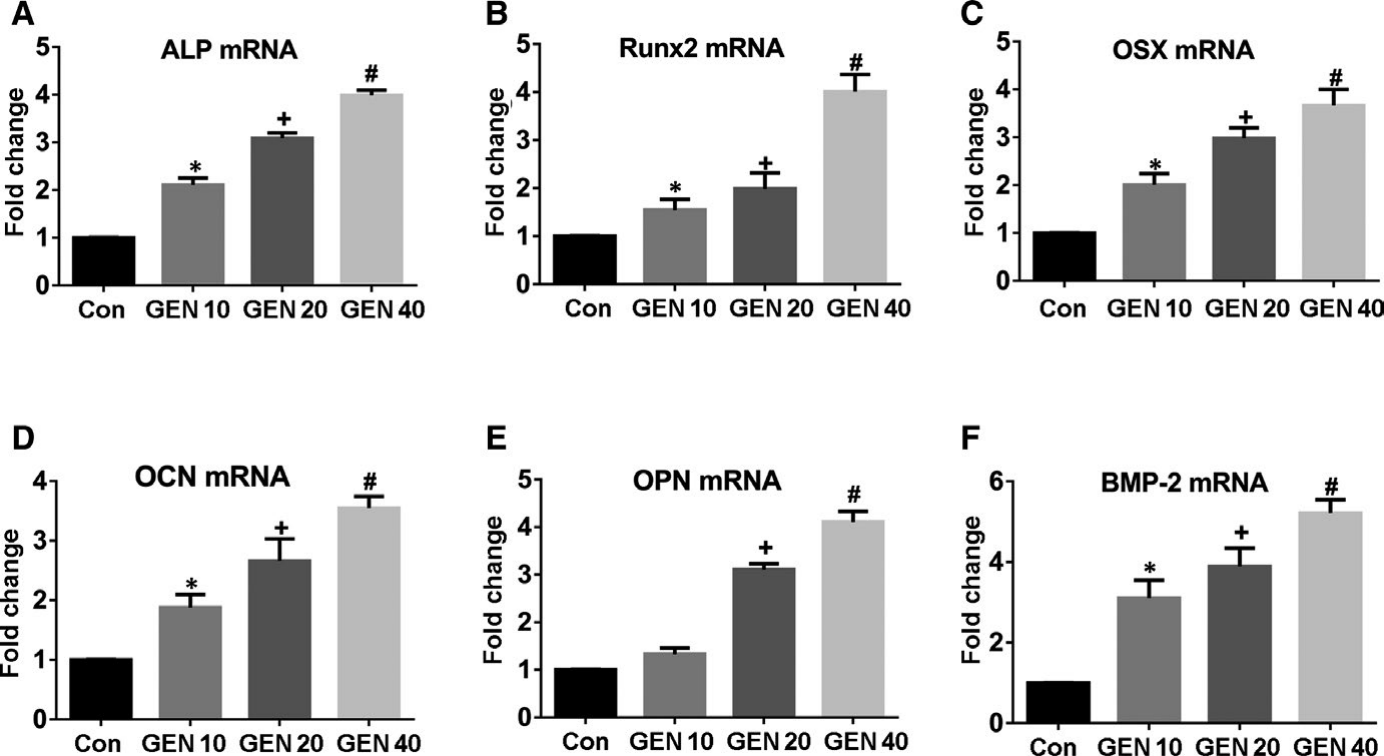
GEN increased the production of ossific‐related genes in vitro. BMSCs were cultivated with GEN (0, 10, 20 and 40 μM) in the condition of osteogenic induction for 2 weeks. (A‐F) mRNA levels of *ALP*, *Runx2*, *OSX*,*OCN*, *OPN* and *BMP2* were detected by q‐PCR analysis. *P <.05 versus control group, ^+^ *P* <.01 versus 10 μM GEN, ^#^ *P* <.001 versus 20 μM GEN

**FIGURE 3:**
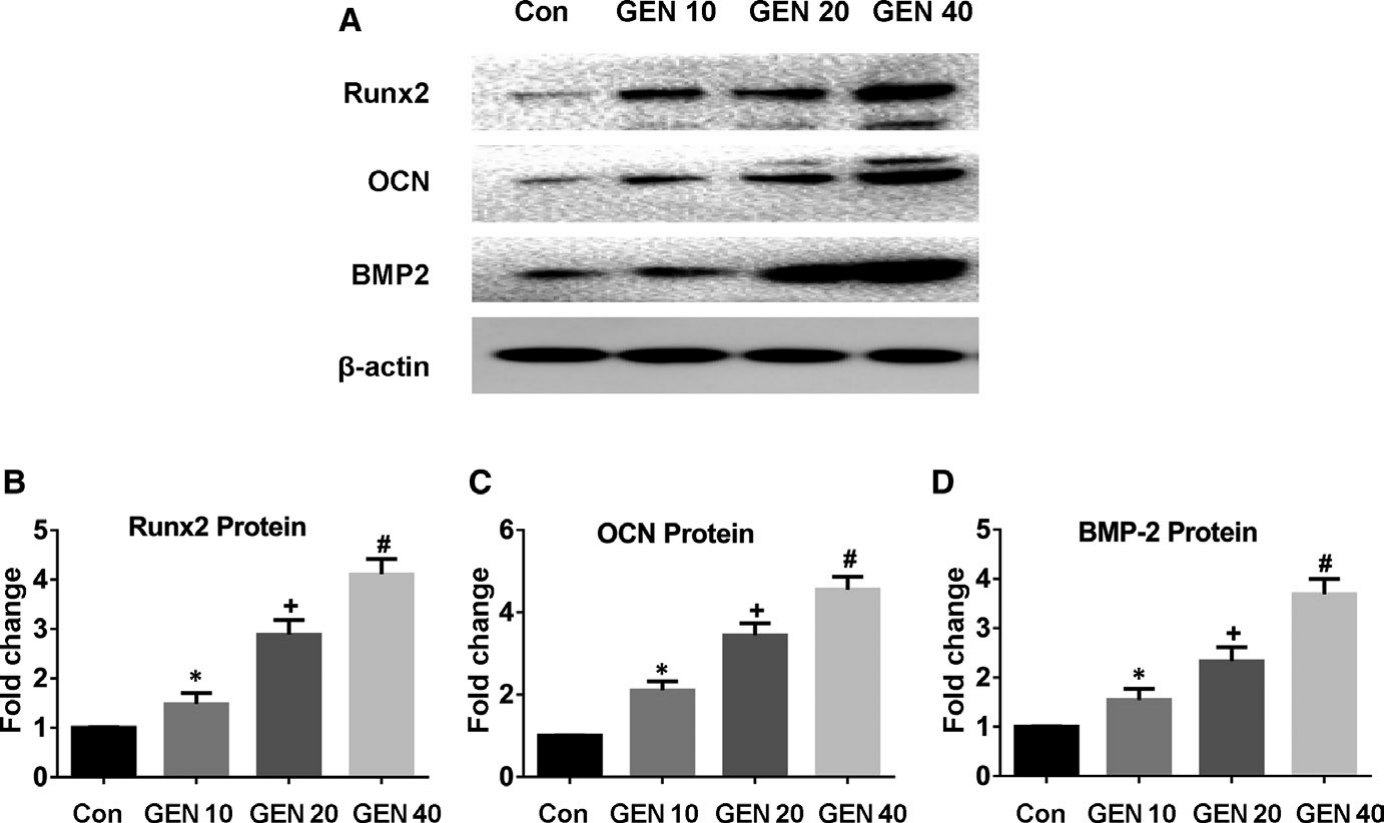
GEN increased the production of osteogenic‐related proteins in vitro. BMSCs were cultivated with GEN (0, 10, 20 and 40 μM) in the condition of osteogenic induction for 2 weeks. Protein level of Runx2 (A, B), OCN (A, C), and BMP2 (A, D) was detected by Western blot. *P <.05 versus group without GEN, ^+^ *P* <.01 versus 10 μM GEN, ^#^ *P* <.001 versus 20 μM GEN

The effect of GEN alone on BMSCs was tested as first. The osteogenic differentiation of the BMSCs treated with GEN for 14 days without osteogenic medium was not significantly affected (Figure S1) (*P* >.05). Next, the osteogenic differentiation of BMSCs treated with GEN for two weeks under osteogenic induction was explored. The higher concentration of GEN, the higher ALP activity (Figure 1C, E) (less than 4‐fold, *P* <.05) and the number of mineralized nodules (Figure 1D, F) (less than 3‐fold, *P* <.05) compared with the control group. In addition, the mRNA expression of the osteogenic genes (ALP, Runx2, OSX, OCN, OPN and BMP2) was significantly increased by GEN, and the increasing trend was concentration‐dependent (Figure 2A‐F)(nearly 4‐fold for ALP and Runx2, less than 4‐fold for OSX and OCN, more than 4‐fold for OPN, and nearly 5‐fold for BMP2, *P* <.05). Similarly, the expression of osteogenic proteins (Runx2, OCN and BMP2) was also promoted by GEN and reached a peak at 40 μM (Figure 3A‐D)(nearly 4‐fold for Runx2, less than 5‐fold for OCN, less than 4‐fold for BMP2, *P* <.05). Therefore, our results revealed that GEN strengthened the osteogenic differentiation in BMSCs.

### 3.3 GEN promotes bone formation in OVX mice

The OVX mouse model was used to confirm these in vitro results. The OVX osteoporosis mouse model is the most used animal model in studying postmenopausal osteoporosis. Ovariectomy can cause bone loss acceleration and cortical bone formation reduction, which are closely related to oestrogen deficiency.^29^, ^37^ To test the effect of the ovariectomy, the body weight and the mass of the uterus were measured (Figure S2)(*P* <.05). The body weight of the OVX group and OVX + GEN group was greater than that of the Sham group (Figure S2A) (less than 1‐fold, *P* <.05). In contrast, the mass of the uterus in the OVX group and OVX + GEN group was less than that in the Sham group (Figure S2B) (less than 3‐fold, *P* <.05).

**FIGURE 4:**
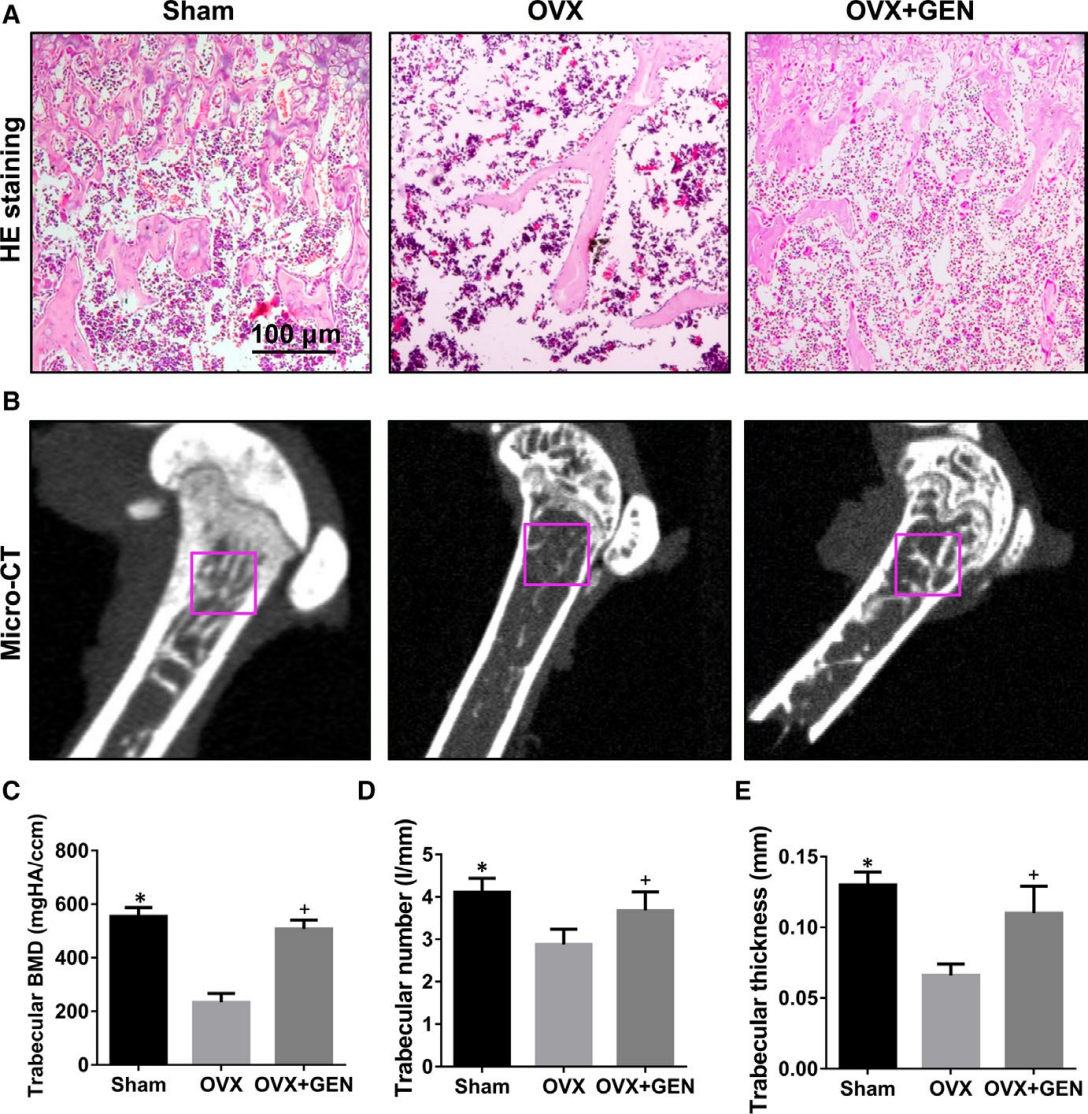
GEN promotes bone formation in OVX mice. (A) After 3 months of GEN treatment, the sections of mice femurs were evaluated by HE staining. (B) Micro‐CT photographs of the statistical analysis site of femur. (C‐E) Statistical credits of bone mineral density (BMD), trabeculae number and trabeculae thickness in different groups. *P <.05 and ^+^ *P*<.05 versus with OVX group

The histopathological images of all groups (sham group, OVX group, and OVX + GEN group) are shown in Figure 4. HE staining results showed that the number of bone trabeculae in the OVX group was significantly less than that in the Sham group, whereas the number of bone trabeculae in the OVX + GEN group was higher than that in the OVX group, but no significant difference was observed between the Sham group and OVX + GEN group (Figure 4A). The results of micro‐CT showed that the BMD, the number of trabeculae and the thickness of the trabeculae in OVX + GEN group were higher than those in the OVX group, whereas the same parameters in the OVX group were remarkably lower than those in the Sham group (Figure 4B‐E) (*P* <.05). However, no statistical difference was found between OVX + GEN group and Sham group.

**FIGURE 5:**
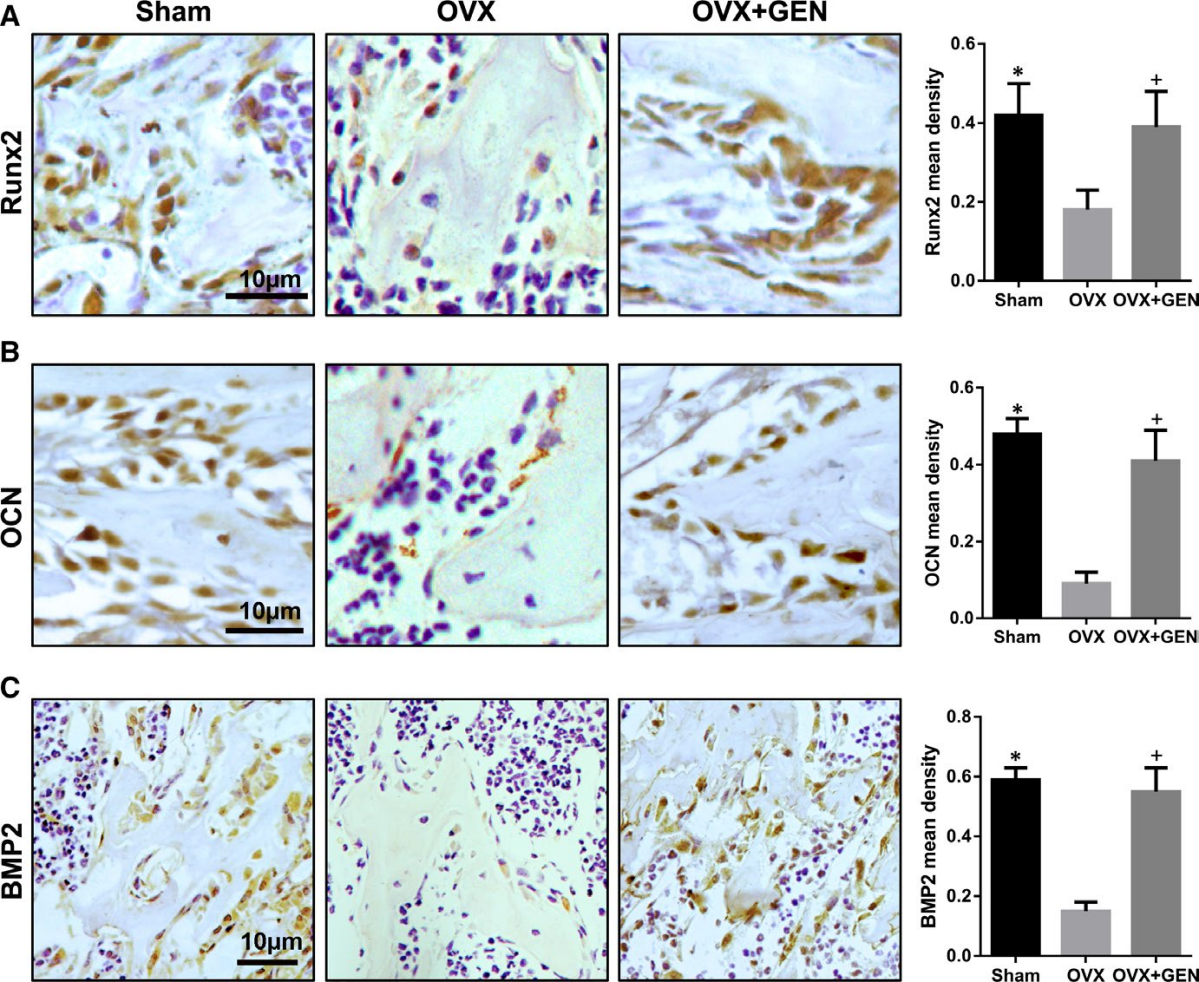
GEN stimulates the expression of osteogenic markers in vivo. Three months after administration of GEN in OVX mice, the level of Runx2 (A), OCN (B), and BMP2 (C) was tested by immunohistochemistry. *P <.05 and ^+^ *P*<.05 versus with OVX group

Subsequently, immunohistochemistry was used to detect the expression of osteogenic proteins (Runx2, OCN and BMP2) in vivo. Runx2 expression in the OVX group was less than that in the Sham group, whereas its expression in the OVX + GEN group was higher than that in the OVX group (Figure 5A) (nearly 2‐fold, *P* <.05). Similar to the results of Runx2, the expression of OCN (Figure 5B) (less than 3‐fold, *P* <.05) and BMP2 (Figure 5C) (nearly 3‐fold, *P* <.05) in OVX group was lower than that in the Sham group and OVX + GEN group. Thus, our results demonstrate that GEN effectively promoted osteogenesis in OVX osteoporotic mice and showed a good anti‐osteoporotic effect.

### 3.4 BMP pathway and Wnt/β‐catenin pathway activated by GEN in BMSCs

**FIGURE 6:**
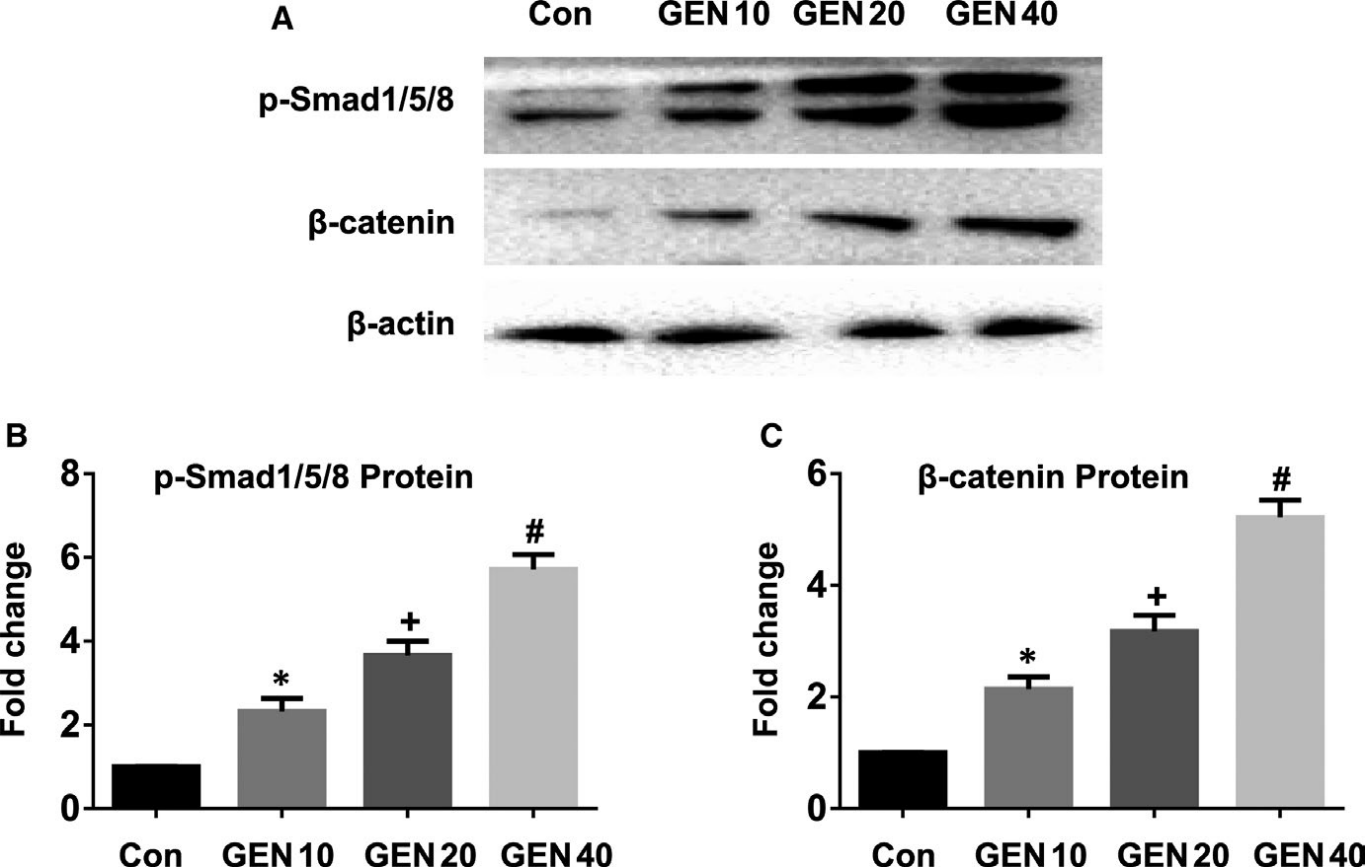
Effect of GEN on BMP signalling and WNT/β‐catenin signalling pathway. BMSCs were cultivated with GEN (0, 10, 20 and 40 μM) in the condition of osteogenic induction for 2 weeks. The protein level of p‐Smad1/5/8 (A, B) and β‐catenin (A, C) was measured by Western blot. *P <.05 versus group without GEN, ^+^ *P* <.01 compared with 10 μM GEN, ^#^ *P* <.001 compared with 20 μM GEN

**FIGURE 7:**
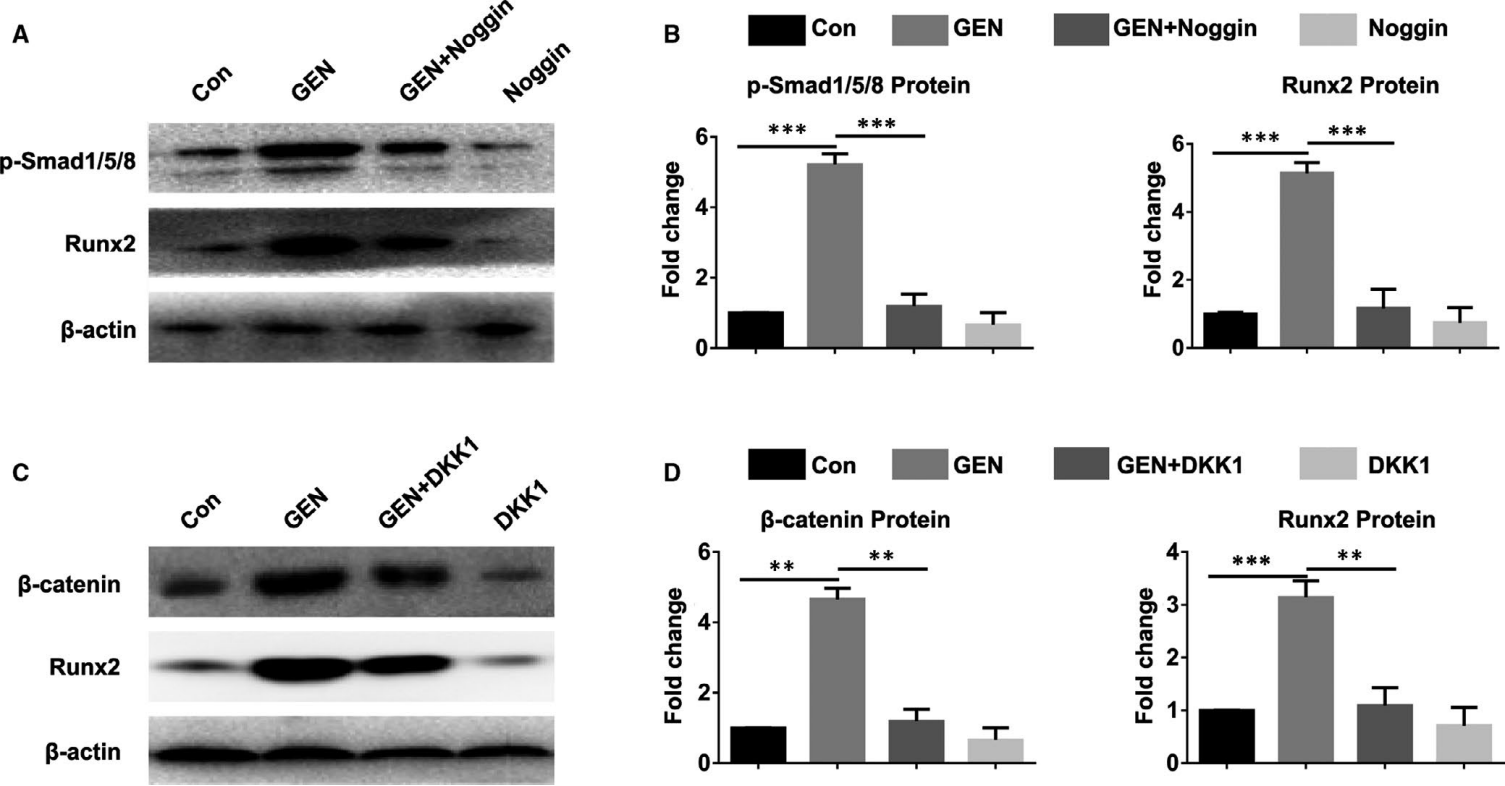
Inhibition of BMP signalling and WNT/β‐catenin signalling pathway blocked GEN‐induced osteogenic differentiation in BMSCs. BMSCs were treated with GEN (40 μM) under osteogenic condition in the presence of Noggin or DKK1 for 2 weeks. The level of p‐Smad1/5/8 (A, B), β‐catenin (C, D), and Runx2 (A‐D) was evaluated by Western blot. **P <.01, ***P <.001

As the BMP signalling ^38^ and WNT signalling ^39^ pathways are related to osteogenesis, the consequence of GEN treatment on the signalling of BMP and WNT/β‐catenin was evaluated. Without osteogenic induction, GEN did not alter the expression of p‐Smad1/5/8 and β‐catenin in BMSCs (Figure S3)(*P* >.05). Next, the effect of GEN on the osteogenic mechanism in BMSCs was further explored. After the treatment of BMSCs with GEN for 2 weeks under osteogenic conditions, the results showed that GEN up‐regulated the expression of p‐Smad1/5/8 and β‐catenin in a dose‐dependent manner (Figure 6A, B, C) (less than 6‐fold for p‐Smad1/5/8, nearly 5‐fold for β‐catenin, *P* <.001). However, the osteogenesis‐potentiating effect of GEN on p‐Smad1/5/8 and β‐catenin was abolished by the treatment with the BMP pathway inhibitor Noggin (Figure 7A‐B) *(P* <.001) and the Wnt/β‐catenin pathway inhibitor DKK1 (Figure 7C‐D) (*P* <.001). Therefore, our results reveal that GEN promoted the osteogenic differentiation via BMP signalling and WNT/β‐catenin signalling.

### 3.5 GEN‐induced osteogenic differentiation is a β‐catenin‐BMP2‐dependent effect

**FIGURE 8:**
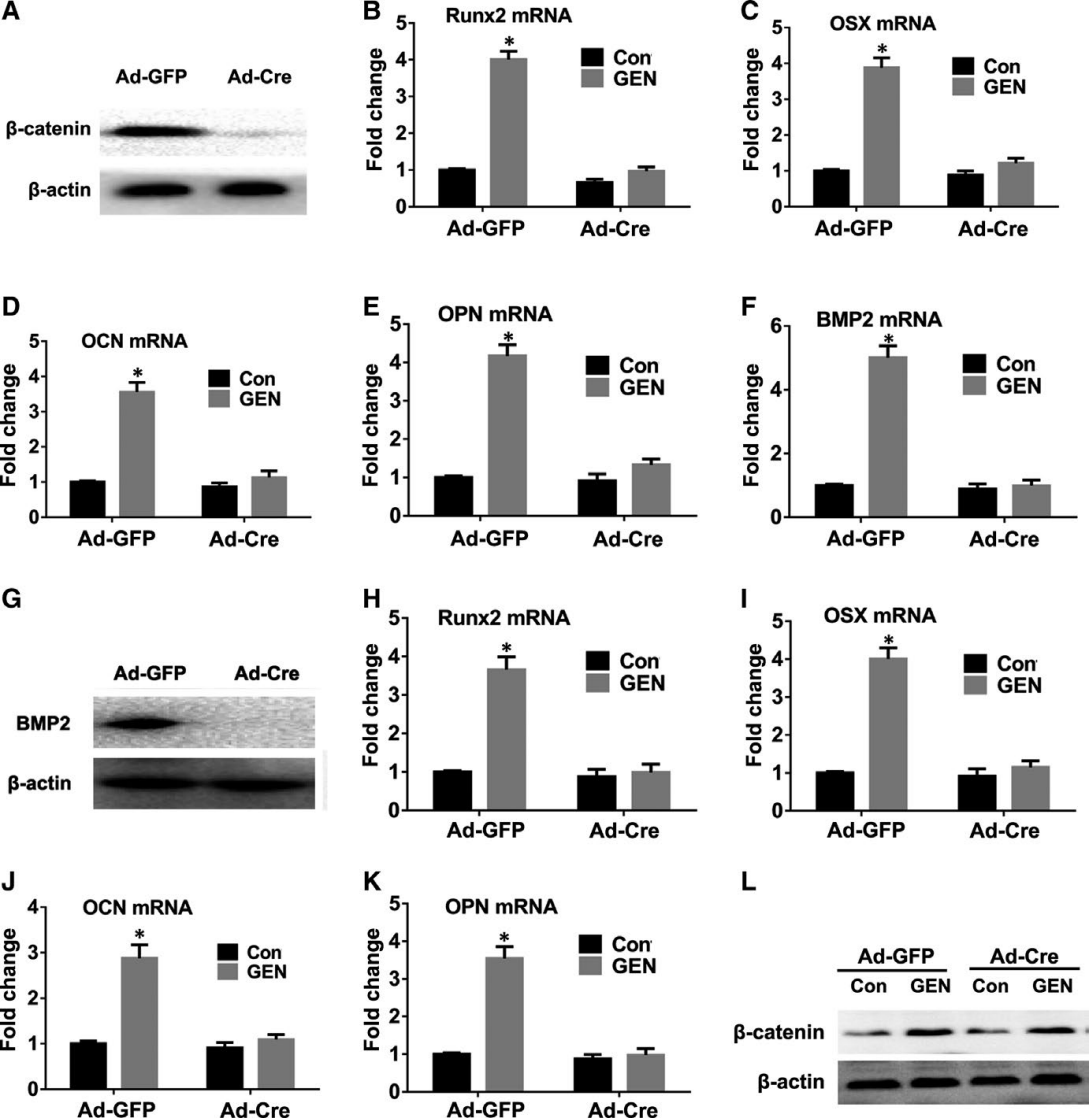
GEN promotes osteogenic differentiation of BMSCs in a β‐catenin‐BMP2‐dependent manner. BMSCs were transfected with either Ad‐GFP or Ad‐Cre, which were used to delete the gene of β‐catenin. Then, the treated cells were incubated with GEN (40μM) for 7 days. The protein expression of β‐catenin (A) was evaluated by Western blot. The mRNA level of *Runx2* (B), *OSX* (C), *OCN* (D), *OPN* (E) and *BMP2* (F) was examined by q‐PCR. BMSCs were transfected with either Ad‐GFP or Ad‐Cre, which were used to delete the gene of BMP2. Then, the treated cells were incubated with GEN (40μM) for 7 days. The protein expression of BMP2 (G) and β‐catenin (L) was estimated by Western blot. The mRNA level of *Runx2* (H), *OSX* (I), *OCN* (J) and *OPN* (K) was tested by q‐PCR. *P <.01 compared with control (Con) group

To further reveal the specific mechanism of GEN in regulating of BMP signalling and Wnt/β‐catenin signalling, gene silencing was performed in vitro. The transfection with Ad‐Cre efficiently silenced the β‐catenin (Figure 8A) and BMP2 (Figure 8G) gene in BMSCs. β‐catenin silencing in BMSCs significantly inhibited the increase of *Runx2*, *OSX*, *OCN*, *OPN* and BMP2 induced by GEN (Figure 8B‐F)(nearly 4‐fold for Runx2 and OSX, less than 4‐fold for OCN, more than 4‐fold for OPN, nearly 5‐fold for BMP2, *P* <.001). In addition, the increase of *Runx2*, *OSX*, *OCN* and *OPN* (Figure 8H‐K) (less than 4‐fold for Runx2, nearly 4‐fold for OSX, less than 3‐fold for OCN, more than 3‐fold for OPN, *P* <.001) induced by GEN was inhibited by silencing the BMP2 gene, although the expression of β‐catenin was not affected (Figure 8L). Our data revealed that the silencing of β‐catenin gene prevented GEN‐mediated up‐regulation of BMP2, although GEN‐induced β‐catenin enhancement was not influenced by silencing the BMP2 gene. The above results indicated that GEN first activates the β‐catenin pathway and then the BMP2 pathway. Therefore, GEN strengthened the osteogenic ability of BMSCs through the β‐catenin‐BMP2 signalling pathway.

## 4 DISCUSSION

GEN is considered an effective drug in the treatment of osteoporosis by preventing the formation of osteoclast.^28^ The inhibition of osteoclast or the activation of osteogenesis exerts a significantly protective effect on osteoporosis.^40^ The current study demonstrated for the first time the effect and potential mechanism of GEN in the osteogenic process of BMSCs both in vitro and in vivo. BMSCs were used to explore the effect of GEN in osteogenesis in vitro. GEN did not show any toxicity on BMSCs at the doses used. In addition, GEN enhanced the expression of ALP and improved the mineralized nodules, increased the expression of osteogenic factors in a concentration‐dependent manner. Finally, the in vivo results showed that GEN accelerated the osteoid formation and mineralization in the mouse femur. All these results indicated that GEN could stimulate osteogenesis in vitro and in vivo. Our results showed that GEN strengthened the osteogenic ability of BMSCs in vitro by the up‐regulation of the β‐catenin‐BMP2 signalling pathway and stimulated bone ossification in vivo. Therefore, GEN could be potentially considered a novel compound in regulating bone metabolism, as it could not only promote osteogenesis but also inhibit bone absorption.

In this study, GEN was found to be able to activate osteogenic differentiation in BMSCs under osteogenic medium. However, there was no effective influence on BMSCs without osteogenic condition. These results suggest that GEN has no significant effect on normal bone metabolic activity, but can promote bone formation in an osteogenic environment. ALP, Runx2 and OSX are the markers of the early stage of osteogenesis, whereas OCN, OPN and BMP2 are the markers of the middle and late stages of osteogenesis.^41^ Here, treating BMSCs with GEN for 14 days could obviously promote ALP expression and mineralized nodule formation. Meanwhile, the osteogenic genes mRNA expression of ALP, Runx2, OSX,OCN, OPN and BMP2 were elevated in a dose‐dependent manner. Moreover, the expression of osteogenic proteins (Runx2, OCN and BMP2) was significantly enhanced in vitro and in vivo. Therefore, these data indicate that GEN has a promoting effect in all stages of osteogenesis and that GEN is a highly promising potential osteogenic drug.

BMP pathway and Wnt pathway occupy a critical position in determining the direction of osteogenesis in BMSCs.^42^, ^43^ The activation of the BMP pathway induces the phosphorylation of Smad1/5/8. Then, the phosphorylated Smad1/5/8 bind to Smad4 and move into the nucleus, thus activating downstream factors of the BMP pathway.^44^ In our study, 14 days of stimulation of BMSCs with GEN increased the expression of p‐Smad1/5/8 in a concentration‐dependent manner, suggesting that GEN could activate the BMP2 pathway during osteogenesis promotion. Additionally, the Wnt/β‐catenin signalling is critical in the therapy of osteoporosis, as it has a significant impact on promoting osteogenesis and regulating bone metabolism.^45^ When the Wnt/β‐catenin signalling is up‐regulated, it can effectively promote the transformation of the precursor osteoblasts into osteoblasts, so as to actively regulate the formation of new bone and improve the structure of the bone itself.^46^ After Wnt pathway activation, the β‐catenin is efficiently translocated into the nucleus, thus stimulating the production of downstream factors.^47^GEN increased the expression of β‐catenin in a dose‐dependent manner, which suggested that GEN could activate both the BMP2 pathway and the Wnt/β‐catenin pathway in BMSCs. It also demonstrated that BMP2 pathway inhibitor Noggin,^48^ and WNT /β‐catenin pathway inhibitor DKK1,^49^ significantly inhibited GEN‐induced osteogenesis in BMSCs, which further indicated that GEN played a critical role in osteogenesis through the BMP2 pathway and the WNT /β‐catenin pathway. Therefore, GEN stimulated the osteogenic ability of BMSCs through both the two signalling pathways.

To reveal the sequential order in the activation of the BMP pathway and Wnt pathway by GEN, β‐catenin or BMP2 was silenced in vitro, resulting in a suppression of GEN‐mediated osteogenesis after the silencing of both genes. In addition, the silencing of β‐catenin completely suppressed GEN‐mediated BMP2 expression, whereas the silencing of BMP2 could not influence GEN‐mediated β‐catenin expression in BMSCs. Thus, BMP2 was a target factor in the downstream of the β‐catenin pathway in BMSCs. Overall, our results demonstrated that GEN stimulated the ossification of BMSCs by the activation of β‐catenin‐BMP2 pathway. The results are consistent with previous studies,^50^, ^51^, ^52^ which revealed that the β‐catenin is a upstream factor of BMP2 signalling.

**FIGURE 9:**
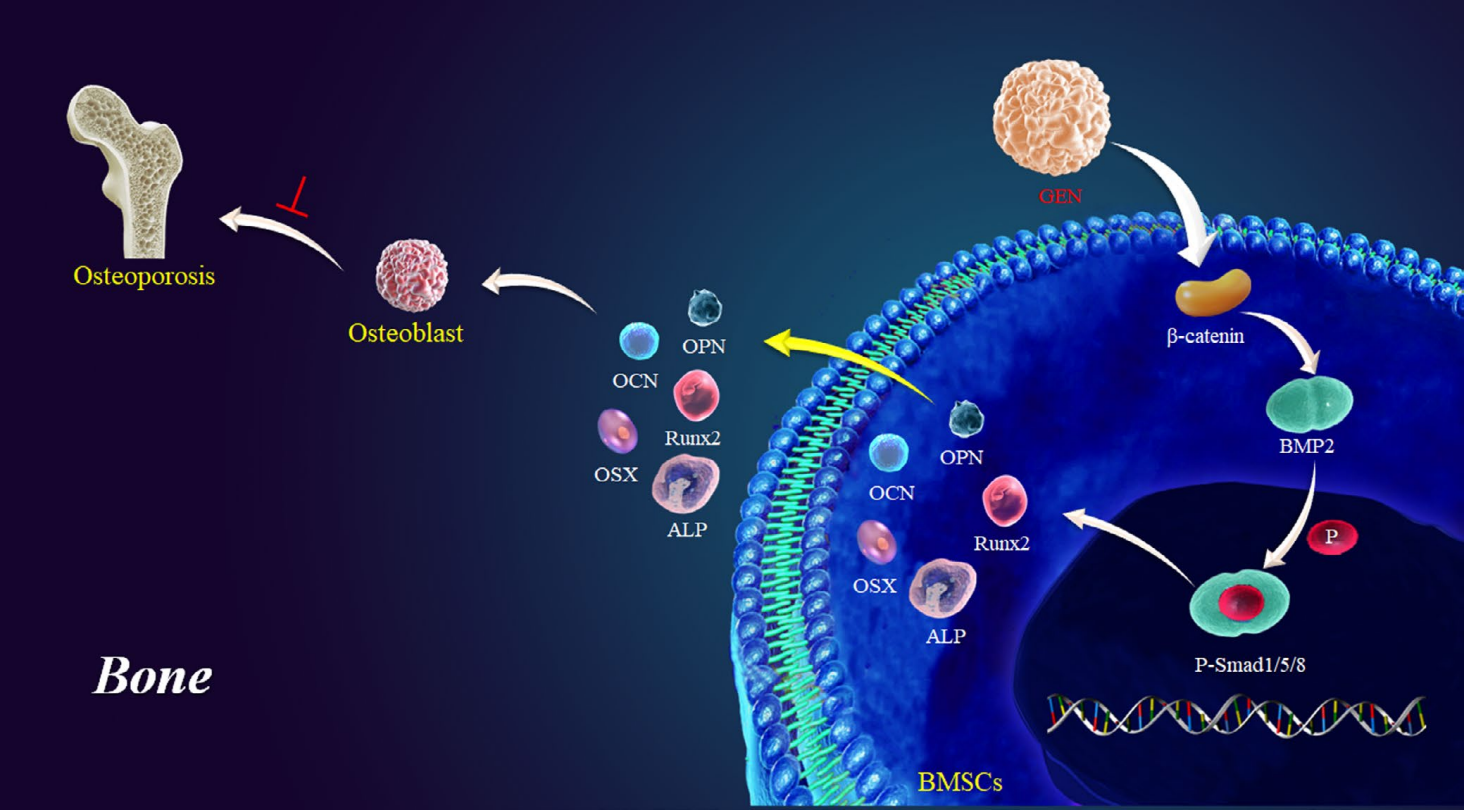
Proposed model depicting the underlying mechanisms of GEN in promoting the osteogenic differentiation of BMSCs

Taken together the results in the current study, a model illustrating the potential mechanism used by GEN to promote the osteogenic differentiation of BMSCs could be proposed. GEN stimulates osteogenesis by increasing ALP, Runx2, OSX, OCN and OPN through the activation of the Wnt/β‐catenin‐BMP2 signalling, thereby promoting the differentiation of BMSCs into osteoblasts (Figure 9).

This study has some limitations. The effect of GEN on human BMSCs from healthy persons and osteoporosis patients would be further to identify our findings.

## 5 CONCLUSIONS

In conclusion, this study provided novel insights into the effect of GEN on BMSCs osteogenic differentiation and its protective effect against bone loss. Although further studies are required to confirm these results, GEN might represent a promising approach in the treatment of osteoporosis.

## CONFLICT OF INTEREST

All authors declare that they have no conflict of interest.

## AUTHOR CONTRIBUTION

**Huaji Jiang:** Conceptualization (equal); Investigation (lead); Writing‐original draft (equal); Writing‐review & editing (equal). **Jialiang Zhong:** Formal analysis (equal); Investigation (equal). **Wenjun Li:** Formal analysis (equal); Investigation (equal). **Jianghui Dong:** Data curation (equal); Formal analysis (equal); Writing‐original draft (equal); Writing‐review & editing (equal). **Cory Xian:** Formal analysis (equal); Writing‐original draft (equal); Writing‐review & editing (equal). **Yung‐Kang Shen:** Data curation (equal); Formal analysis (equal). **Lufeng Yao:** Data curation (equal); Formal analysis (equal). **Qiang Wu:** Writing‐original draft (equal); Writing‐review & editing (equal). **Liping Wang:** Conceptualization (lead); Writing‐original draft (lead); Writing‐review & editing (lead).

## Supporting information

Fig S1

Fig S2

Fig S3
