# Supplementary figures and images for: Gentiopicroside promotes the osteogenesis of bone mesenchymal stem cells by modulation of β‐catenin‐BMP2 signalling pathway
Source: J Cell Mol Med. 2021 Nov 15;25(23):10825–36. doi: 10.1111/jcmm.16410 (PMC8642693; doi:10.1111/jcmm.16410)

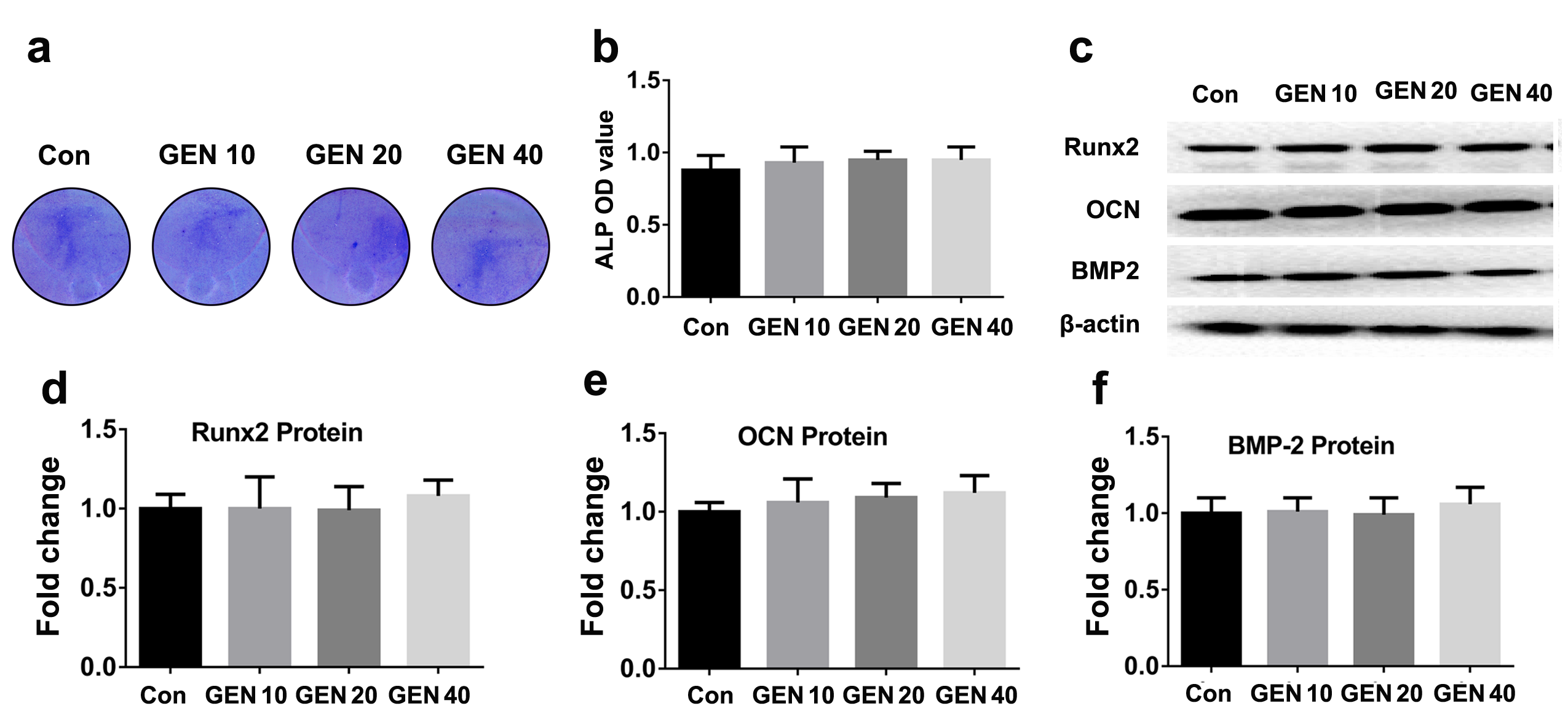

Supplement: Supplementary file 1 — Fig S1 [file JCMM-25-10825-s002.tif]

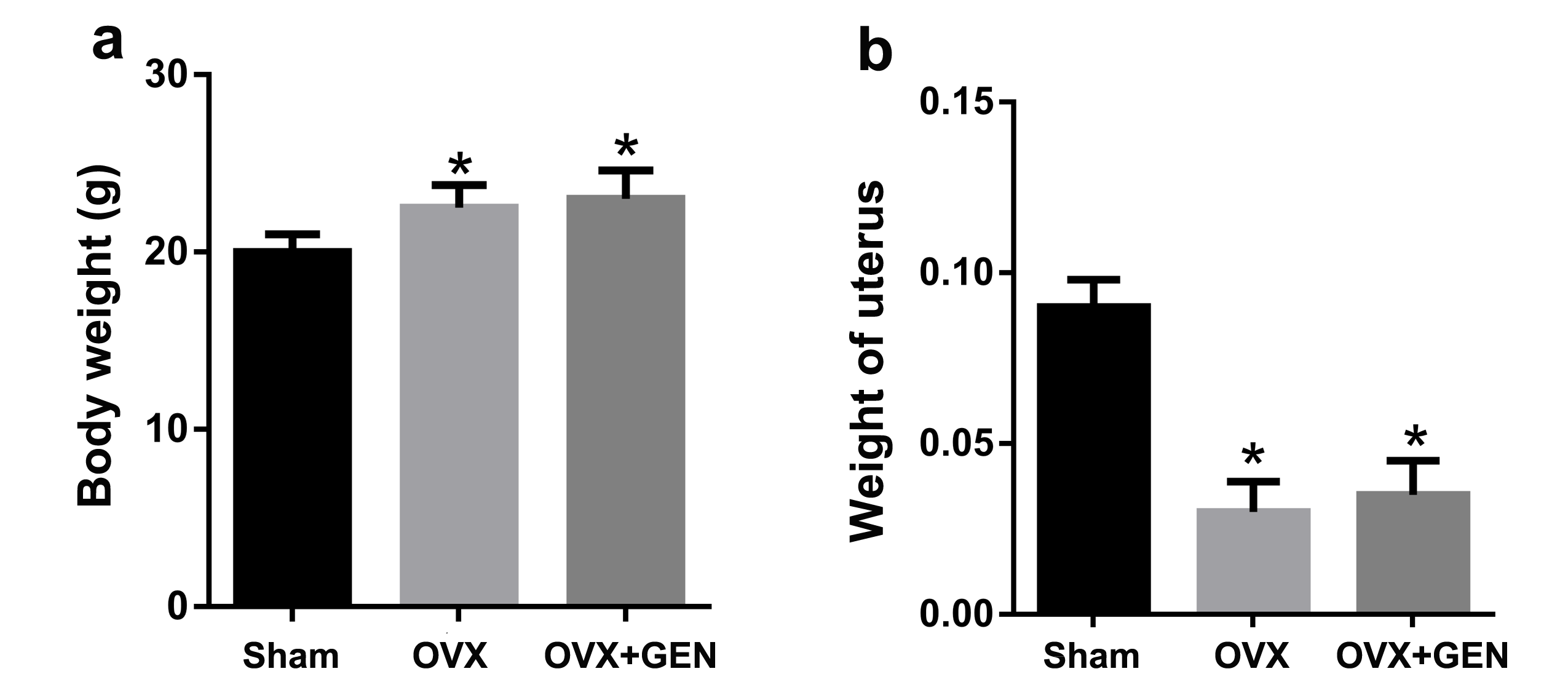

Supplement: Supplementary file 2 — Fig S2 [file JCMM-25-10825-s001.tif]

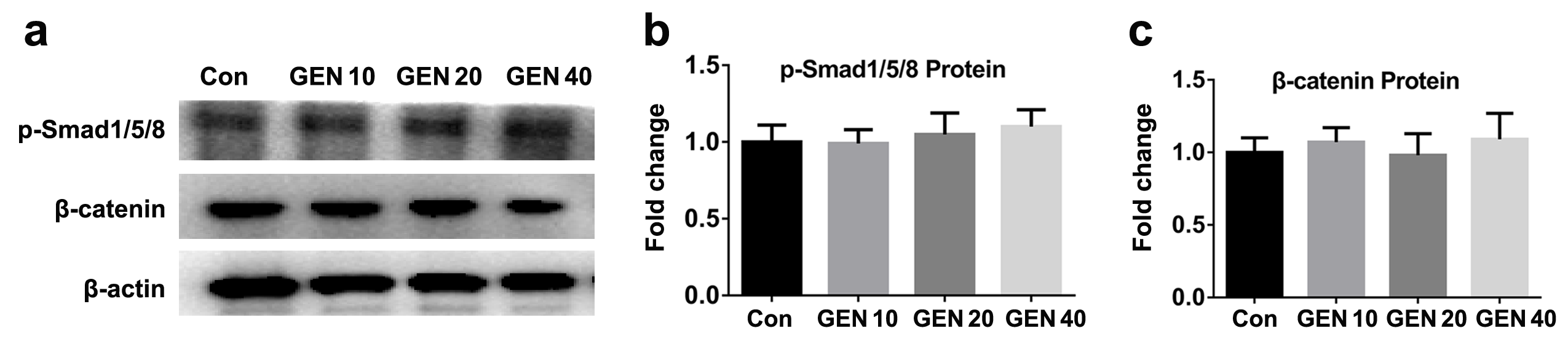

Supplement: Supplementary file 3 — Fig S3 [file JCMM-25-10825-s003.tif]
